# Promoting caste equality in the labor market: The role of self-confidence

**DOI:** 10.1371/journal.pone.0327299

**Published:** 2025-07-31

**Authors:** Qiqi Wang, Tushi Baul, Sujoy Chakravarty, Tanya Rosenblat

**Affiliations:** 1 School of Economics, Xi’an University of Finance and Economics, Xi’an, Shaanxi, China; 2 Instagram, Menlo Park, California, United States of America; 3 School of Social Sciences, Jawaharlal Nehru University, New Delhi, India; 4 School of Information, University of Michigan, Ann Arbor, Michigan, United States of America; Duke University, UNITED STATES OF AMERICA

## Abstract

We study how people perceive the self-confidence of individuals from different castes in India. In an experimental Indian labor market where employers and workers belong to different castes, employers evaluate worker resumes to predict the future productivity of workers who perform a real effort task. The baseline group uses resumes that reveal a productivity signal i.e.- performance in a practice task and caste information, while the treatment group receives an additional measure of worker self-confidence. We find that employers in both groups exhibit a discriminatory wage differential against lower caste workers. However, employers in the treatment group weigh lower caste workers’ self-confidence more heavily than that of higher caste workers. This differential effect of confidence compensates for the lower evaluation and hence wage given to lower caste workers due to discrimination. From a policy perspective, these findings highlight the importance of non-cognitive skill training, such as training sessions for employment interviews where applicants can signal their self-confidence through interaction with employers.

## 1 Introduction

India’s caste system has contributed to various socioeconomic disparities between higher and lower caste groups [1–3]. In particular, these two groups differ significantly in self-confidence [3–5]. Since self-confidence plays an important role in shaping economic behavior and determining economic outcomes [6–14], part of the persistent gap in economic outcomes between higher and lower caste groups in India is associated with the variation in self-confidence levels across different caste groups [4, 5].

While the existing literature focuses on differences in self-confidence among caste groups, little is known regarding how employers perceive the self-confidence of different castes. In this study, we posit that self-confidence expressed by individuals is a non-cognitive attribute that may exacerbate or mitigate discrimination towards these individuals by members of other groups. For example, if self-confidence is rewarded more for higher caste workers compared to lower caste workers then signaling self-confidence in the job market can undermine the welfare of lower caste individuals and further widen the caste gap. On the other hand, if lower caste workers derive higher returns from self-confidence than higher caste workers then displaying self-confidence to employers can lead to a narrowing of the caste wage gap. Therefore, examining employers’ perception of self-confidence of different castes offers a deeper understanding of the caste gap in labor market outcomes.

In this study, we conduct a series of laboratory experiments in a labor market setting to investigate how higher and lower caste workers’ self-confidence is perceived by employers. Our research yields three key findings. First, lower caste workers are subject to systematic discrimination by employers. Second, employers recognize the self-confidence of lower caste workers more than that of higher caste workers. Third, this caste difference in perception of self-confidence offsets the wage differentials resulting from discrimination.

The experiment proceeds as follows. Initially, there is a worker stage in which participants play the role of a worker. Workers first solve puzzles in a one-minute practice period. Then, they are incentivized to provide an evaluation of own productivity in a following five-minute employment period. This self-evaluation measures the worker’s self-confidence. Finally, the worker solves puzzles in the employment period in which he or she receives piece rate payment from the experimenter.

After the worker stage, the experiment switches to the employer stage in which other participants play the role of employer. An employer receives a mini-resume displaying a worker’s labor market characteristics. The information shown on the resume depends on the treatment. In the baseline treatment, the resume shows the worker’s practice performance and caste identity. In the confidence treatment, the resume displays practice performance, caste identity, and self-confidence. The employer observes the resume and predicts the worker’s performance in the employment period. This evaluation of future productivity determines the wage paid to the worker.

We observe caste discrimination as employers assign lower evaluations to lower caste workers compared to higher caste workers with similar resume characteristics. The results show evidence of caste discrimination in both treatments. In the baseline treatment, a consistent gap exists between evaluations for higher caste and lower caste workers. In the confidence treatment, the evaluation gap widens as workers’ practice performance increases.

However, we find that when worker self-confidence is made available, employers strongly respond to it. As a worker’s confidence level increases, so does their evaluation. Interestingly, the results indicate that employers attribute more value to lower caste workers’ self-confidence to that of higher caste workers. Thus, workers from lower caste backgrounds receive higher evaluations than their higher caste counterparts with the same level of self-confidence. Furthermore, this differential treatment on self-confidence results in the evaluation gap due to discrimination decreasing as worker self-confidence increases. At the average level of self-confidence, a large part of the caste gap due to discrimination is eliminated.

When discussing the type of discrimination in our experiment, we first argue that it is unlikely to be taste based discrimination [15]. In the Confidence treatment, employers condition their evaluations on workers’ practice performance and self-confidence. This indicates that employers use both cognitive and non-cognitive information and do not evaluate workers solely based on identity. It is also unlikely to be statistical discrimination [16, 17]. The two pieces of information available to employers–practice performance and self-confidence–do not differ across caste groups. Therefore, employers cannot use these observable measures to infer group-level differences or to distinguish workers based on caste.

We posit that the observed discrimination is likely driven by stereotypes. In this view, perceived inter-group differences arise not from objective reality, but from mental models or stereotypes about the abilities of individuals from different groups [18, 19]. Stereotypes also help explain how self-confidence is recognized and interpreted by employers. These perceptions may be rooted in longstanding attitudes and practices embedded in the caste system. Most notably, the imposition of norms related to social and occupational hierarchy has resulted in lower caste groups historically facing systemic disadvantages in various spheres of life. These include education, health, access to credit markets, entrepreneurial opportunities and labor markets [20–27]. Consequently, individuals from lower caste backgrounds may have to face more obstacles and overcome more hurdles to attain similar levels of confidence as their higher caste counterparts. Our results suggest that employers recognize this difference in societal and economic barriers resulting from historical under privilege for lower caste communities. Accordingly, they acknowledge that the latter may have had to realize higher efforts to develop a level of self-confidence similar to that of higher caste individuals. As a result of this, they value the self-confidence of a lower caste worker more than that of a higher caste worker.

Our paper contributes to the existing literature by demonstrating that the disclosure of self-confidence benefits lower caste workers and reduces the caste gap in labor market outcomes. There are two primary strands of research related to self-confidence and economic outcomes. The first of these examines group differences, such as caste or gender, in self-confidence [3–5, 7, 8, 10, 11, 13], and obtains that lower caste individuals and women tend to have lower levels of self-confidence. The second strand of literature investigates the positive impact of self-confidence on economic behavior and outcomes [6–14]. By combining these two strands of research, one might logically conclude that signaling self-confidence in the labor market exacerbates the caste gap against lower caste workers. However, our study counterintuitively shows that though overall it may be true that individuals from underprivileged communities display lower confidence, employers and evaluators place greater importance on their self-confidence.

Our research offers new policy implications for addressing caste disparities. Traditionally, the main affirmative action policy that has been employed by the Indian government to promote caste equity provides lower castes with reservations in higher education, central government jobs, and national legislatures [23, 28–30]. This centralized and structural provision has over time led to somewhat improved socioeconomic outcomes for underprivileged castes [31, 32]. However, studies in different contexts have revealed unintended consequences of affirmative action in the form of quotas, including negative stereotyping and stigmatization of beneficiary groups [33], exacerbation of inter-group animosity [34], distorted reviews and sabotage from peers and increased difficulties in identifying high-ability beneficiaries [35–37]. Our study proposes an alternative, independent, decentralized approach to facilitate caste equality in the labor market. This approach can be implemented by itself or in conjunction with other existing policies of affirmative action. We emphasize the need to prioritize the development of non-cognitive skills, particularly for individuals that hail from more underprivileged socioeconomic contexts. Our findings indicate that when applicants communicate their self-confidence during the job application process, individuals from higher and lower caste backgrounds receive statistically indistinguishable evaluations from employers. This finding implies that the ability to project self-confidence in interviews can play a critical role in fostering caste wage equality. To this end, we propose the implementation of training programs that focus on enhancing interview techniques, enabling candidates to actively exhibit their self-confidence during the interview process. Such programs will lead to more equitable assessments by employers and, as a result, promote caste equality within the workplace, in spite of the pre-existing biases against lower caste individuals.

The rest of the paper is organized as follows. Section 2 provides a background for this study. Section 3 introduces experimental design and data. Section 4 reports and discusses the results. Section 5 concludes.

## 2 Background

### 2.1 The classification of castes

Currently, there is a four-fold division of categories of the Indian population with respect to caste - General Category comprising upper or forward caste individuals, and those belonging to less privileged communities classified as from Scheduled Castes (SC), Scheduled Tribes (ST), and Other Backward Classes (OBC). According to the Census (2011), they respectively constitute 35.6%, 16.2%, 8.2% and 40% of the total population. From the 1980s, The Indian constitution have set reservation quotas of 15% and 7.5% in government jobs and educational institutions for SC and ST categories, respectively. In 2011, 27% reservation was implemented for OBCs. In this study, we categorize the participants from the General Category as higher caste individuals, and participants from the SC, ST, and OBC categories as lower caste individuals.

### 2.2 Caste disparities in economic outcomes

Individuals belonging to underprivileged castes realize lower economic outcomes as compared to those belonging to forward castes [22, 38, 39]. Studies indicate that there is lower presence of underprivileged castes in Indian research institutes [27], higher incidence of health issues such as hypertension among tribals [25], lower access to institutional loans [24], lower ownership of and earnings from business enterprises [20, 21, 26], and weaker family, social and cultural networks [40, 41] for lower caste individuals. In particular, Madheswaran and Attewell [42] study the wage gap between upper and lower caste candidates in the urban labor market, and find that the wages of SC/STs are 15% lower than equally qualified general category individuals.

Caste discrimination is considered an important factor leading to caste disparities in economic outcomes [43]. For example, Thorat and Attewell [44] use a correspondence study and find that job applicants with Dalit or Muslim names on average receive significantly fewer call backs from private sector companies as compared to similarly qualified candidates with higher caste Hindu names. Similarly, Siddique [45] finds that on average lower caste applicants need to send 20 percent more resumes than higher caste applicants to receive similar callbacks. Banerjee *et al*. [46] perform a callback experiment where they send fictitious resumes out for job openings, and find that business process outsourcing (BPO) firms favor upper caste applicants.

Caste difference in self-confidence is shown to be another important factor contributing to the caste gap in economic outcomes. Deshpande and Newman [40] use a longitudinal survey of upper and lower caste university students in Delhi and find that in spite of having similar training and credentials, lower caste students have lower expectations and consider themselves in disadvantaged positions as compared to upper caste students. Dasgupta *et al*. [3] combine rich data from incentivized tasks and surveys conducted among a large sample of university students in a Seemingly Unrelated Regression (SUR) framework and find that those from historically marginalized communities fare worse than upper castes along several dimensions of behavior such as competitiveness and confidence and personality traits such as grit, locus of control, and conscientiousness. Hoff and Pandey [4] study the effect of caste-based stereotype threat on the real effort task performance of children participants. They find that when caste identity is made publicly salient, performance and piece-rate earnings in maze solving tasks among lower caste children is lower than that of their upper caste counterparts as compared to a control group in which caste identity is not made public.

### 2.3 The relationship between self-confidence and economic outcomes

Various research studies have shown that self-confidence is an important indicator for economic outcomes. For example, non-cognitive skills such as the expression of self-confidence or the display of resilience and grit are seen to lead to individuals ceteris-paribus realizing superior outcomes [14, 47]. Kamas and Preston [11] use laboratory tasks to show that women with a taste for competition and higher self-confidence obtain earnings which are no different from men. Moreover, these earnings are higher than those of other women. More generally, Page and Ruebeck [14] use a longitudinal field experiment on children who were 8–11 years old at inception. Controlling for measured ability, they find 22 years later that higher childhood confidence predicts higher adolescent test scores, high school and college graduation, majoring or working in STEM, earnings and job retention. In a similar vein, Chen *et al*. [10] find that men who are confident of their quantitative abilities have higher earnings. Dargnies *et al*. [12] manipulate the self-confidence of their experimental subjects in a laboratory labour market experiment using hard or easy tasks. They observe that the relatively under-confident accept early offers made by employers before observing their task performance, rather than opt to be in a later stage where they may potentially receive higher wages.

## 3 The experiment

### 3.1 Experimental design

**Fig 1:**
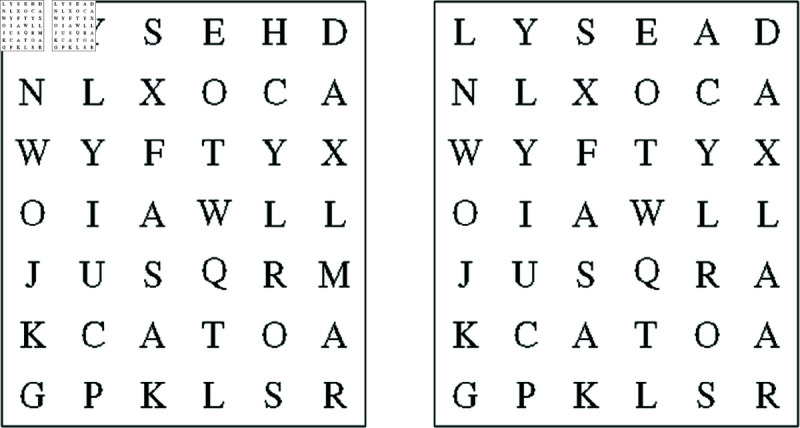
The character puzzle.

We simulate a labor market where participants coming from different castes play roles of “employer” and “worker.” Employers evaluate productivity and set the wage of workers who perform a real-effort task. The task involves solving character puzzles containing 7 rows and 6 columns of Latin alphabets. Fig 1 shows an example of a typical puzzle. In the right-hand side box, there are two random positions in which the letters differ from those in the left-hand side box. To solve the puzzle, workers need to identify these two letters. As participants solve these by visual inspection and comparison, performance is determined solely by effort and not by linguistic or analytical abilities. The same type of character games have been used in Mobius and Rosenblat [6] and Mobius *et al*. [48] to study discriminatory wage differentials in other labor settings.

For each worker, there are three periods: A warm-up period with two non-payoff relevant puzzles, a one-minute practice period, and a five-minute employment period. Even though workers are not financially compensated in the practice period, they are incentivized to perform well because they are informed that their wage will be set by employers based on their practice performance. In the employment period, workers receive 50 tokens per puzzle solved correctly.

To elicit a worker’s self-confidence, we adopt the methodology of Mobius and Rosenblat [6]. After the practice period but before entering the employment period, workers are informed of their practice performance and answer the following question: *How many puzzles do you believe that you can solve in the following employment period?* The answer serves as our measure of the worker’s self-confidence regarding performance in the puzzle task. Workers are rewarded with 40 tokens if their answers match their actual performance. A similar absolute measure of self-confidence in a real effort task has also been employed by Dasgupta *et al*. [1].

It must be noted that our measure of self-confidence is similar to the concept of self-efficacy in the social psychology literature. The social cognitive theory of psychology has defined self-efficacy as one’s belief in his or her ability to succeed in specific situations or accomplish tasks [49]. According to Bandura [49] and Bandura [50], self-confidence refers to the strength of belief, which may or may not be accompanied by self-efficacy. For example, self-confidence could manifest in an agent being confident that he will fail at a task. Technical differences aside, self-confidence and self-efficacy generally move in the same direction, where belief in one’s agentive capabilities and the strength of that belief reinforce each other.

For an employer, the task is to evaluate some workers’ productivity in the employment period. Every employer evaluates 10 randomly selected workers. For each evaluated worker, the employer receives 300 tokens. However, every unit of mis-calibrated evaluation reduces employer earnings by 10 tokens.

**Fig 2:**
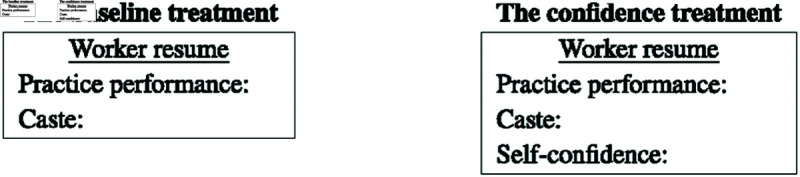
Resume treatments.

To help employers with evaluation, we construct a mini-resume of each worker and present it to the employer. The information on the resume depends on the treatment. In the baseline treatment, the resumes contain the worker’s practice performance and caste. In the confidence treatment, we add the worker’s self-confidence to the resume. Examples of the resumes are given in Fig 2.

Finally, employer evaluations set the wage of the workers. In addition to other payments, the worker receives 50 tokens per unit of average employer evaluation. At the end of the experiment, both the workers and employers fill out a survey. We collect demographics including caste information through the survey.

Studying the effect of worker self-confidence on employer decisions is challenging given limited opportunities to observe worker-employer interactions in empirical data from organizations. Our experimental methodology offers a unique advantage in examining these interactions. Specifically, we explicitly measure a worker’s self-confidence via her belief in her ability to solve a real-effort task. We then present this self-confidence metric, as well as a signal of cognitive ability, for employers to evaluate worker ability. This evaluation process simulates worker-employer interactions in a real labor market and enables us to isolate the effect of self-confidence on employer evaluations. Using this approach, we analyze the extent to which employers recognize worker self-confidence in making employment decisions.

### 3.2 Experimental data

We conduct the experiment with students in a large public university in India with a nationally representative student body. We recruit a total of 267 students from science, humanities, social sciences and economics majors. Participants who are all above the age of 18 give their consent in writing, using an informed consent form framed according to the guidelines of the Institutional Review Board (IRB) of New York University, Abu Dhabi. The period in which we recruit subjects and conduct our experiments is from the 1st of May to the 15th of August 2015.

The role of the worker is randomly assigned to 81 participants. The remaining 186 participants are employers. We assign more participants to the Confidence treatment since in this treatment we introduce our key explanatory variable: self-confidence. The larger sample size helps us more precisely estimate its effect. Caste information of participants is obtained through a post-experiment survey. Table 1 summarizes the caste composition of workers and employers.

**Table 1:** Numbers of participants.

	Workers	Employers	
		Baseline treatment	Confidence treatment
Higher caste	45	40	43
Lower caste	36	40	63
Total	81	80	106

The experiment is conducted on paper forms. Worker sessions are conducted separately at a time earlier than employer sessions. Both worker and the employer sessions take about one hour. At the end of the experiment, tokens earned by participants are exchanged for cash at the rate of five tokens to one Indian Rupee (INR). The total earnings of each participant include a participation fee of INR 100 and earnings from playing in the role of a worker or employer. On average each participant earns about INR 530 from the experiment. The average earning per participant is approximately USD 28 in terms of purchasing power parity (PPP).

Table 2 presents the summary statistics of worker performance. During the practice period, higher caste and lower caste workers on average solve 3.5 and 3.3 puzzles, respectively. With respect to self-confidence, higher caste and lower caste workers predict before the employment period that they can solve 20.0 and 19.3 puzzles, respectively. These differences are not statistically significant. However, during the employment period, higher caste workers actually solve 18.8 puzzles while lower caste workers solve 15.9 puzzles. This productivity gap of 2.9 puzzles is statistically significant at the 5% level.

**Table 2:** Summary statistics of worker performance.

	Workers			
	All	Higher caste	Lower caste	Difference
	(1)	(2)	(3)	(2) vs. (3)
Performance in the practice period	3.4	3.5	3.3	0.2
	(1.5)	(1.2)	(1.8)	(0.3)
Self-confidence	19.5	20.0	19.3	0.7
	(7.9)	(8.4)	(7.3)	(1.8)
Performance in the employment period	17.5	18.8	15.9	2.9^**^
	(5.1)	(5.0)	(4.9)	(1.1)
Number of workers	81	45	36	

*Notes*: (i). Standard deviations are in parentheses in columns (1)-(3). (ii). For differences, standard errors are in parentheses. (iii). Significance levels of 10%, 5%, and 1% are denoted by ^*^, ^**^, and ^***^, respectively.

Table 3 presents employer evaluations. As seen in column (1), in the confidence treatment, pooled over all workers, the average employer evaluation is 18.7 puzzles. When we divide workers based on their caste groups in columns (2) and (3), we observe that employers assign 19.0 puzzles for higher caste workers and 18.0 puzzles for lower caste workers. This evaluation difference of 1.0 puzzle is statistically significant at the 1% level.

**Table 3:** Summary statistics of employer evaluations.

	Workers			
	All	Higher caste	Lower caste	Difference
	(1)	(2)	(3)	(2) vs. (3)
Confidence treatment	18.7	19.0	18.0	1.0^***^
	(2.9)	(3.6)	(3.3)	(0.3)
Baseline treatment	18.8	19.1	18.2	0.9^**^
	(3.2)	(3.8)	(3.6)	(0.4)

*Notes*: (i). Standard deviations are in parentheses in columns (1)-(3). (ii). For differences, standard errors are in parentheses. (iii). Significance levels of 10%, 5%, and 1% are denoted by ^*^, ^**^, and ^***^, respectively.

For the baseline treatment, the average employer evaluation is 18.8 puzzles. For higher caste workers, the average employer evaluation is 19.1 while for lower caste workers, it is 18.2. This productivity difference of 0.9 puzzles is statistically significant at the 5% level.

## 4 Results

In this section, we begin by presenting evidence of caste-based discrimination. Then, we investigate how employers perceive the self-confidence of the various caste groups. Lastly, we examine how employers’ differential recognition of workers’ self-confidence reduces the caste evaluation gap attributable to discrimination.

### 4.1 Evidence of caste discrimination

We define caste discrimination as employers assigning lower evaluations to lower caste workers than to higher caste workers with similar resume characteristics. To look for the presence of caste discrimination, we construct the regression model below for the confidence treatment:

$$Evaluation_{ij}\;=\alpha_{0}+\alpha_{1}LowerCaste_{i}+\alpha_{2}Practice_{i}+\alpha_{3}Practice_{i}\cdot LowerCaste_{i}\;+\alpha_{4}Confidence_{i}+\alpha_{5}Confidence_{i}\cdot LowerCaste_{i}+E_{j}+\sigma_{ij},$$ (1)

where ${Evaluation}_{ij}$ is employer *j*’s evaluation for worker *i*, *Practice* is the normalized value of worker’s performance during the practice period with zero mean and unit standard deviation, *Confidence* is the normalized value of worker’s self-confidence with zero mean and unit standard deviation, *LowerCaste* is a dummy variable that describes the worker’s caste identity (LowerCaste=0 for higher caste workers and LowerCaste=1 for lower caste workers), and *E* is a fixed effect accounting for different average evaluations across employers.

As a robustness check, we estimate all regressions using the raw measures of *Practice* and *Confidence*. The regression results are shown in the supporting tables. The main findings remain similar with one exception. In S2 and S4 Tables specifically, the variable *LowerCaste* demonstrates significantly larger standard errors when the interaction terms are included, compared to the specifications without the interaction terms. In contrast, in Tables 5 and 7, the standard errors of the variable *LowerCaste* exhibit consistent patterns between specifications with and without the interaction terms.

In Eq 1, the coefficient $\alpha_{2}$ represents the impact of a one standard deviation change in higher caste workers’ practice performance on employer evaluations. The coefficient $\alpha_{3}$ measures the differential effect of lower caste workers’ practice performance in comparison to that of higher caste workers. The overall effect of lower caste workers’ practice performance is defined as $\alpha_{2}+\alpha_{3}$. In the absence of discrimination, where higher caste and lower caste workers’ practice performances have identical effects on the employer evaluations, we expect that $\alpha_{3}=0$. Otherwise, it indicates caste discrimination as employers assign different weights to higher caste and lower caste workers’ practice performance.

The estimation results, as presented in column (1) of Table 4, show that a one standard deviation increase in higher caste workers’ practice performance is associated with a $\alpha_{2}=4.598$ puzzle increase in employer evaluations. The estimate of $\alpha_{3}$ suggests that lower caste workers’ practice performance has a significantly smaller effect than higher caste workers’, with a gap of 1.265 puzzles. As a result, the overall effect of lower caste workers’ practice performance is just 3.333 puzzles. This means that employers assign different weights to practice performance based on caste, and predict an evaluation gap between higher caste and lower caste workers with the same level of practice performance. Moreover, this evaluation gap becomes wider as the level of practice performance increases. When we break down the employers based on caste group in columns (2) and (3), the results suggest that both higher caste and lower caste employers place less emphasis on lower caste workers’ practice performance.

**Table 4:** Regression analysis of employer evaluations in the confidence treatment.

Dependent variable	*Evaluation*					
Employers	All	Higher caste	Lower caste	All	Higher caste	Lower caste
	(1)	(2)	(3)	(4)	(5)	(6)
*LowerCaste*	–0.257	–0.467	–0.098	–0.118	–0.336	0.025
	(0.231)	(0.330)	(0.316)	(0.231)	(0.335)	(0.314)
*Practice*	4.598^***^	4.954^***^	4.255^***^	3.787^***^	4.100^***^	3.581^***^
	(0.216)	(0.280)	(0.318)	(0.128)	(0.184)	(0.176)
Practice·LowerCaste	–1.265^***^	–1.517^***^	–0.977^**^			
	(0.267)	(0.365)	(0.382)			
*Confidence*	1.338^***^	1.065^***^	1.566^***^	1.764^***^	1.518^***^	1.915^***^
	(0.166)	(0.229)	(0.234)	(0.125)	(0.181)	(0.170)
Confidence·LowerCaste	0.831^***^	1.059^***^	0.608^*^			
	(0.255)	(0.361)	(0.354)			
${\text{R}}^{2}$	0.567	0.672	0.503	0.559	0.662	0.497
*N*	1046	423	623	1046	423	623

*Notes*: (i). N is number of evaluations. (ii). Standard errors are in parentheses. (iii). Significance levels of 10%, 5%, and 1% are denoted by ^*^, ^**^, and ^***^, respectively.

Result 1: Employers in the confidence treatment discriminate against lower caste workers, resulting in a caste gap that expands as workers’ practice performance increases.

In the baseline treatment, employers evaluate worker resumes based on practice performance and caste identity, without observing their self-confidence level. This treatment serves two purposes. First, it tests the robustness of caste discrimination when self-confidence is not observable. Second, if the variable *Confidence* is not statistically or economically significant for employer evaluations, it confirms that our self-confidence measure is not correlated with other worker attributes.

Table 5 presents the estimation results for the baseline treatment. In column (1), the coefficient of *LowerCaste* is significantly negative. This indicates that employers in the baseline treatment also exhibit caste discrimination against lower caste workers in their evaluations. To see whether the evaluation gap varies across different levels of practice performance, we examine the coefficient of the interactions term Practice·LowCaste in column (4). The insignificance of the coefficient suggests that, unlike the confidence treatment, the evaluation gap in the baseline treatment remains constant across different levels of practice performance.

**Table 5:** Regression analysis of employer evaluations in the baseline treatment.

Dependent variable	*Evaluation*					
Employers	All	Higher caste	Lower caste	All	Higher caste	Lower caste
	(1)	(2)	(3)	(4)	(5)	(6)
*LowerCaste*	–0.473^**^	–0.647^*^	–0.278	–0.473^**^	–0.645^*^	–0.271
	(0.226)	(0.356)	(0.277)	(0.226)	(0.357)	(0.277)
*Practice*	5.523^***^	5.213^***^	5.806^***^	5.533^***^	5.193^***^	6.029^***^
	(0.115)	(0.179)	(0.141)	(0.187)	(0.279)	(0.241)
Practice·LowerCaste				–0.015	0.033	–0.314
				(0.222)	(0.349)	(0.277)
*Confidence*	0.070	–0.069	0.177	0.069	–0.064	0.170
	(0.117)	(0.187)	(0.143)	(0.119)	(0.193)	(0.143)
${\text{R}}^{2}$	0.614	0.541	0.701	0.614	0.541	0.701
*N*	789	399	390	789	399	390

*Notes*: (i). N is number of evaluations. (ii). Standard errors are in parentheses. (iii). Significance levels of 10%, 5%, and 1% are denoted by ^*^, ^**^, and ^***^, respectively.

It is important to note that the variable *Confidence* is both statistically and economically insignificant. This shows that caste discrimination persists in this treatment where self-confidence is not displayed to employers. This insignificance also confirms that our measure of worker self-confidence is not correlated with other factors.

In columns (2) and (3), we separate the employers based on their caste group. Both higher caste and lower caste employers assign lower evaluations to lower caste workers. The caste gap in higher caste employers’ evaluations is statistically significant at the 10% level.

Result 2: Employers in the baseline treatment discriminate against lower caste workers, resulting in a constant evaluation gap between higher and lower caste workers.

### 4.2 Employers’ differential recognition of worker self-confidence

In Eq 1, the coefficient $\alpha_{4}$ captures the effect of higher caste workers’ self-confidence on employer evaluations, while the coefficient $\alpha_{5}$ measures the differential effect of self-confidence on employer evaluations between lower caste and higher caste workers. The effect of self-confidence on employer evaluations for lower caste workers is therefore $\alpha_{4}\;+\;\alpha_{5}$. If there is no caste difference in acknowledging self-confidence, we expect that $\alpha_{5}=0$. Otherwise, employers’ recognition for higher caste and lower caste workers’ self-confidence is different.

According to the results presented in column (1) of Table 4, the coefficient $\alpha_{4}$ for higher caste workers’ self-confidence indicates that if the self-confidence of higher caste workers increases by one standard deviation, employer evaluations increase by 1.338 puzzles. The estimate for $\alpha_{5}$ suggests that the confidence effect of lower caste workers is stronger than that of higher caste workers. Specifically, for each standard deviation increase in self-confidence, employer evaluations increase by 2.169 puzzles for lower caste workers. Columns (2) and (3) of Table 4 demonstrate that the confidence effect of lower caste workers is stronger than that of higher caste workers, irrespective of the caste group to which the employers belong.

Result 3: Employers assign more weight to lower caste workers’ self-confidence than to that of higher caste workers.

Based on the results presented in Table 4, we can analyze how self-confidence reduces the caste evaluation gap due to discrimination. The estimate of $\alpha_{3}$ in column (1) indicates that, for a one standard deviation increase in practice performance, lower caste workers are evaluated about 1.265 puzzles lower than higher caste workers. On the other hand, the estimate of $\alpha_{5}$ suggests that each standard deviation increase of self-confidence results in 0.831 more puzzle for lower caste workers compared to higher caste workers. Therefore, the provision of self-confidence to employers enables lower caste workers to narrow the evaluation gap and reduce the influence of discrimination.

Result 4: The employer’s differential assessment of worker self-confidence reduces the caste evaluation gap due to discrimination.

We now examine the overall caste gap in employer evaluations controlling for practice performance and self-confidence. Consider the following regression model:

$$Evaluation_{ij}=\beta_{0}+\beta_{1}LowerCaste_{i}+\beta_{2}Practice_{i}+\beta_{3}Confidence_{i}+E_{j}+\epsilon_{ij},$$ (2)

where $\beta_{1}$ measures the overall caste evaluation gap in the confidence treatment.

The results are reported in columns (4)–(6), Table 4. The coefficient of *LowerCaste* in column (4) is both statistically and economically insignificant, indicating that at the overall level, employer evaluations for higher caste and lower caste workers are identical in the confidence treatment. In columns (2) and (3), we separate the employers based on caste group. There is no caste evaluation gap at the overall level for either higher caste or lower caste employers.

Result 5: At the overall level, there is no caste gap in employer evaluations in the confidence treatment.

### 4.3 The treatment effect of self-confidence

We have examined the effect of self-confidence on reducing the caste gap in evaluations. In this section, we test whether presenting self-confidence to employers systematically alter employer evaluations compared to those in the baseline treatment. If the latter were true, some of the effect of confidence that we tabulate in our study could be an artefact of our specific laboratory procedure. Consider the following equation:

$$Evaluation_{ij}=\gamma_{0}+\gamma_{1}LowerCaste_{i}+\gamma_{2}Practice_{i}+\gamma_{3}Treatment_{j}+\mu_{ij},$$ (3)

where Treatment=0 for employers in the baseline treatment and Treatment=1 for employers in the confidence treatment. The treatment effect of observing workers’ self-confidence is measured by the coefficient $\gamma_{3}$. We estimate the equation with standard errors clustered at the employer level.

The results of Eq 3 are reported in Table 6. Column (1) shows that at the experiment level, there is a significant caste gap in employer evaluations disfavoring lower caste workers. The employer evaluations for lower caste workers are on average 0.571 puzzles lower than those for higher caste workers. In addition, workers’ practice performance is an important indicator for employer evaluations. An increase of one standard deviation in practice performance increases employer evaluations by 4.841 puzzles.

**Table 6:** The treatment effect of self-confidence.

Dependent variable	*Evaluation*		
Employers	All	Higher caste	Lower caste
	(1)	(2)	(3)
*LowerCaste*	–0.571^***^	–0.860^**^	–0.331
	(0.219)	(0.330)	(0.289)
*Practice*	4.841^***^	4.868^***^	4.832^***^
	(0.160)	(0.203)	(0.234)
*Treatment*	–0.039	–0.638	0.527
	(0.442)	(0.618)	(0.598)
${\text{R}}^{2}$	0.545	0.574	0.529
*N*	1835	822	1013

*Notes*: (i). N is number of evaluations. (ii). Standard errors are in parentheses. (iii). Significance levels of 10%, 5%, and 1% are denoted by ^*^, ^**^, and ^***^, respectively.

Importantly, the coefficient of the treatment dummy, $\gamma_{3}$, is both economically and statistically insignificant. This means that the visibility of workers’ self-confidence does not systematically shift employer evaluations to a different level. Instead, it only serves to mitigate the evaluation gap between higher caste and lower caste workers.

When dividing the employers according to caste in columns (2)-(3), we find that higher and lower caste employers behave similarly in evaluating workers’ practice performance. The coefficients of practice performance are close to each other for these two groups. The treatment dummies are also insignificant for both higher caste and lower caste employers. However, the caste gap against lower caste workers is generated mainly by higher caste employers. For lower caste employers the caste gap against lower caste workers is negative but statistically insignificant.

Result 6: The disclosure of worker self-confidence does not artefactually alter employer evaluations compared to scenarios where there is no self-confidence information provided.

### 4.4 The actual effect of self-confidence on worker performance

Until this point, we have examined how employers perceive workers’ self-confidence. Now, we analyze the actual impact of self-confidence on worker performance. This analysis provides insights into what employers should do in terms of evaluations. Consider the following regression model:

$$Productivity_{i}=\delta_{0}+\delta_{1}LowerCaste_{i}+\delta_{2}Practice_{i}+\delta_{3}Confidence_{i}+\zeta_{i},$$ (4)

where *Productivity* is the number of puzzles that a worker solves in the employment period.

The coefficient $\delta_{3}$ measures the actual effect of self-confidence on worker performance. If the null hypothesis that $\delta_{3}=0$ cannot be rejected, self-confidence has no effect on productivity. In this case, the confidence effects on employer evaluations are all employer “mistakes". Otherwise, it indicates that self-confidence is indeed a valuable metric for employers to consider.

The results are shown in column (1) of Table 7. The significantly positive estimate of $\delta_{3}$ reveals that a one standard deviation increase in self-confidence leads to an increase of 1.784 puzzles in worker productivity. This means that when employers take self-confidence into account during evaluations, they are aligning with a reliable predictor of performance. This significant confidence effect on productivity also hints that workers possess private knowledge of their abilities that is not fully reflected in their practice performance.

**Table 7:** Regression analysis of worker productivity.

Dependent variable	*Productivity*		
Workers	All		Lower castes
	(1)	(2)	(3)
*LowerCaste*	–2.457^***^	–2.443^***^	
	(0.864)	(0.866)	
*Practice*	1.909^***^	2.715^***^	1.461^**^
	(0.481)	(0.810)	(0.618)
Practice·LowerCaste		–1.253	
		(1.012)	
*Confidence*	1.784^***^	1.491^**^	2.031^**^
	(0.482)	(0.625)	(0.805)
Confidence·LowerCaste		0.540	
		(1.006)	
${\text{R}}^{2}$	0.456	0.467	0.395
*N*	81	81	36

*Notes*: (i). N is number of workers. (ii). Standard errors are in parentheses. (iii). Significance levels of 10%, 5%, and 1% are denoted by ^*^, ^**^, and ^***^, respectively.

In column (2), after introducing interaction terms, the effect of confidence remains significant. It appears that self-confidence has a stronger impact on the productivity of lower caste workers, while practice performance has a weaker impact, although these differential effects are not statistically significant. This suggests that assigning more importance to self-confidence and less to practice performance is likely a more precise approach for evaluating lower caste workers. These results are consistent with earlier findings obtained from the employer regressions in the confidence treatment.

To further verify that self-confidence is an effective predictor of productivity among lower caste workers, we estimate Eq 4 separately for the subsample of lower caste workers. The results are reported in column (3) of Table 7. The coefficient of self-confidence remains significantly positive within the lower caste group. Specifically, a one standard deviation increase in self-confidence predicts an increase of 2.031 puzzles. This further supports the interpretation that self-confidence contains real information value, even within the caste.

### 4.5 The motivation for employers’ caste discrimination

We discuss the potential underlying reasons behind caste discrimination in employers’ evaluations. Three (sets of) theories have been suggested to explain discriminatory practices. According to the model of taste based discrimination by Becker [15], employers with prejudice against a certain group of workers perceive the costs of hiring workers from this target group to be higher than actual wages, leading them to offer lower wages. By contrast, statistical discrimination states that non-prejudiced, profit-maximizing employers use group statistics to discriminate between workers with similar individual characteristics, resulting in wage gaps that are economically efficient and based on true empirically observed group differences [16, 17, 51]. In such a situation, discrimination is the result of a signal extraction problem, and the differential treatment of members of different groups is due to imperfect information [52]. Finally, extending insights from sociology that posit culture as a collection of mental models [53], Demeritt and Hoff [18] and Hoff *et al*. [19] introduce the concept of schematic discrimination. Its defining feature is that it is unconscious or conscious discrimination based on mental models in the form of cultural schemas. In such a situation, perception of group attributes such as productivity or trustworthiness may not be ’objective’, i.e., based on true empirical measurement, but on stereotypes, which are oversimplified mental models triggered by certain factual or fictional exemplars, that are culturally primed. Bordalo *et al*. [54] allude to the availability heuristic [55] to recognize that stereotypes tend to exaggerate attributes of a group that are perceived to be different to other groups, even if these attributes may not be important for the stereotyped group.

We argue that caste discrimination in our experiment is not driven by taste. If that were the case, employers would not condition their evaluations on workers’ self-confidence. On the contrary, we observe that in the confidence treatment, workers’ self-confidence provides informative signals for employers, who use this information to counter discrimination against lower caste workers and reduce the evaluation gap between lower and higher caste workers. This observation provides evidence against taste based discrimination.

To distinguish between statistical discrimination and discrimination based on stereotypes, it is crucial to determine whether employers are aware of actual caste differences in worker skills. In our experiment, there are two direct metrics of worker skills: performances in the practice round and employment period. There is also a task-specific self-confidence measure that is elicited from the workers. Employers can observe practice performance and self-confidence, but task productivity in the employment period is not disclosed to them when they perform their evaluation. Therefore, the distinction between statistical discrimination and stereotypes lies in whether or not there is a caste difference in the variables practice performance and self-confidence that are observed by the employer. Based on the summary statistics presented in Table 2, and the worker productivity regression in Table 7, we find no statistically significant difference in practice performance or confidence between individuals from different castes. Therefore, employers should not strictly be able to perform statistical discrimination when using practice performance or self confidence to differentiate between workers of different castes. However, employers assign lower (higher) evaluations for lower caste workers based on practice score (self-confidence). For this reason, we posit that discrimination in our experiment appears to be primarily driven by caste stereotypes. Nevertheless, as signs on the coefficients for the interaction of practice score and self-confidence with the lower caste category in the worker productivity regression in Table 7 match those from the corresponding interactions in the evaluation regression, we cannot completely rule out statistical discrimination.

## 5 Conclusion

Our study explores whether employer recognition of worker self-confidence varies by worker caste. It shows that employers place significant value on worker self-confidence when this information is available. However, we observe that employers give extra weight to self-confidence of workers from lower castes as compared to workers from higher castes. This differential recognition of self-confidence helps to reduce the caste gap due to discrimination. Consequently, at the average level of self-confidence, lower caste workers earn as much as their higher caste counterparts. These results provide valuable insights into the role of self-confidence in combating discrimination, particularly in the labor market.

It is worth noting that the experimental labor market we utilize in our study follows a one-shot design. It would be interesting to investigate how worker behavior differs in a multi-round evaluation environment. We conjecture that two possible predictions may arise from this scenario. The first prediction argues that once lower-caste workers learn that being confident can decrease the evaluation gap, they may strategically display a greater sense of self-confidence relative to higher-caste workers. The second prediction posits that as lower caste workers do not need to be as self-confident as higher caste workers to receive similar evaluations, they project less self-confidence as compared to their lower caste counterparts.

In a broader context, our experimental framework can be applied to labor market interactions where both employers and workers have social identities such as gender or race. This is particularly relevant in settings where gender or racial stereotypes have limited opportunities. As a consequence, women and disadvantaged races are often underrepresented in high-level positions. Traditionally, promoting gender or racial diversity often involves reserving positions in institutions. However, our work proposes an alternative approach: developing a labor market that values workers’ soft information such as self-confidence. If the self-confidence of disadvantaged groups receives greater acknowledgement, this labor market can establish more equitable evaluations for all groups. The latter would help provide a more level playing field for career advancement, thereby increasing representation of disadvantaged groups in senior-level positions.

## Supporting information

S1 Table Regression analysis of employer evaluations in the confidence treatment. (PNG)

S2 Table Regression analysis of employer evaluations in the baseline treatment. (PNG)

S3 Table The treatment effect of self-confidence on employer evaluations. (PNG)

S4 Table Regression analysis of worker productivity. (PNG)

S1 File Experimental instructions. (PDF)

S2 File Data of workers. (XLS)

S3 File Data of employers. (XLS)

S4 File Inclusivity in global research questionnaire. (DOCX)
