# Supplementary figures and images for: Promoting caste equality in the labor market: The role of self-confidence
Source: PLoS One. 2025 Jul 31;20(7):e0327299. doi: 10.1371/journal.pone.0327299 (PMC12312929; doi:10.1371/journal.pone.0327299)

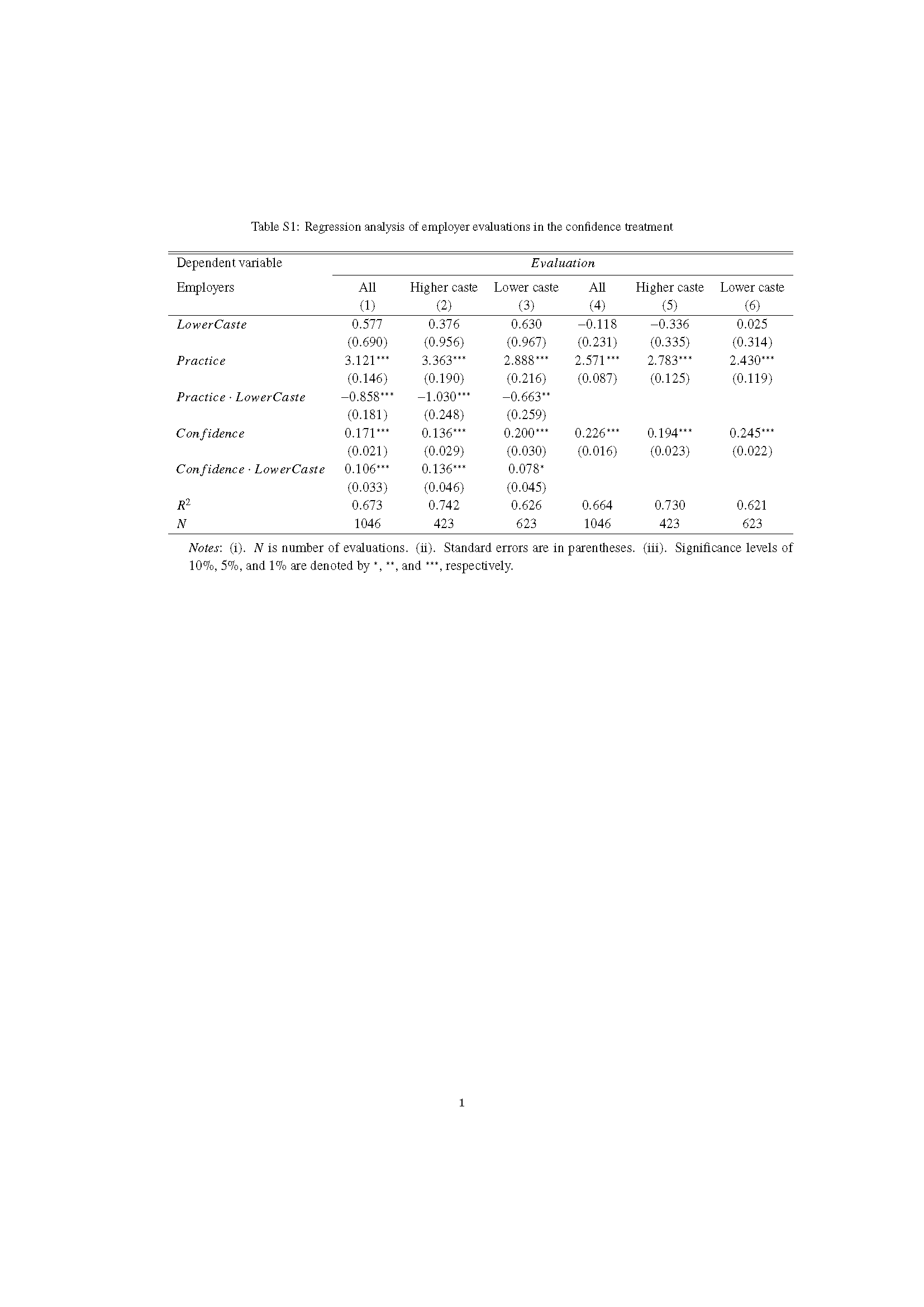

Supplement: S1 Table — (PNG) [file pone.0327299.s001.png]

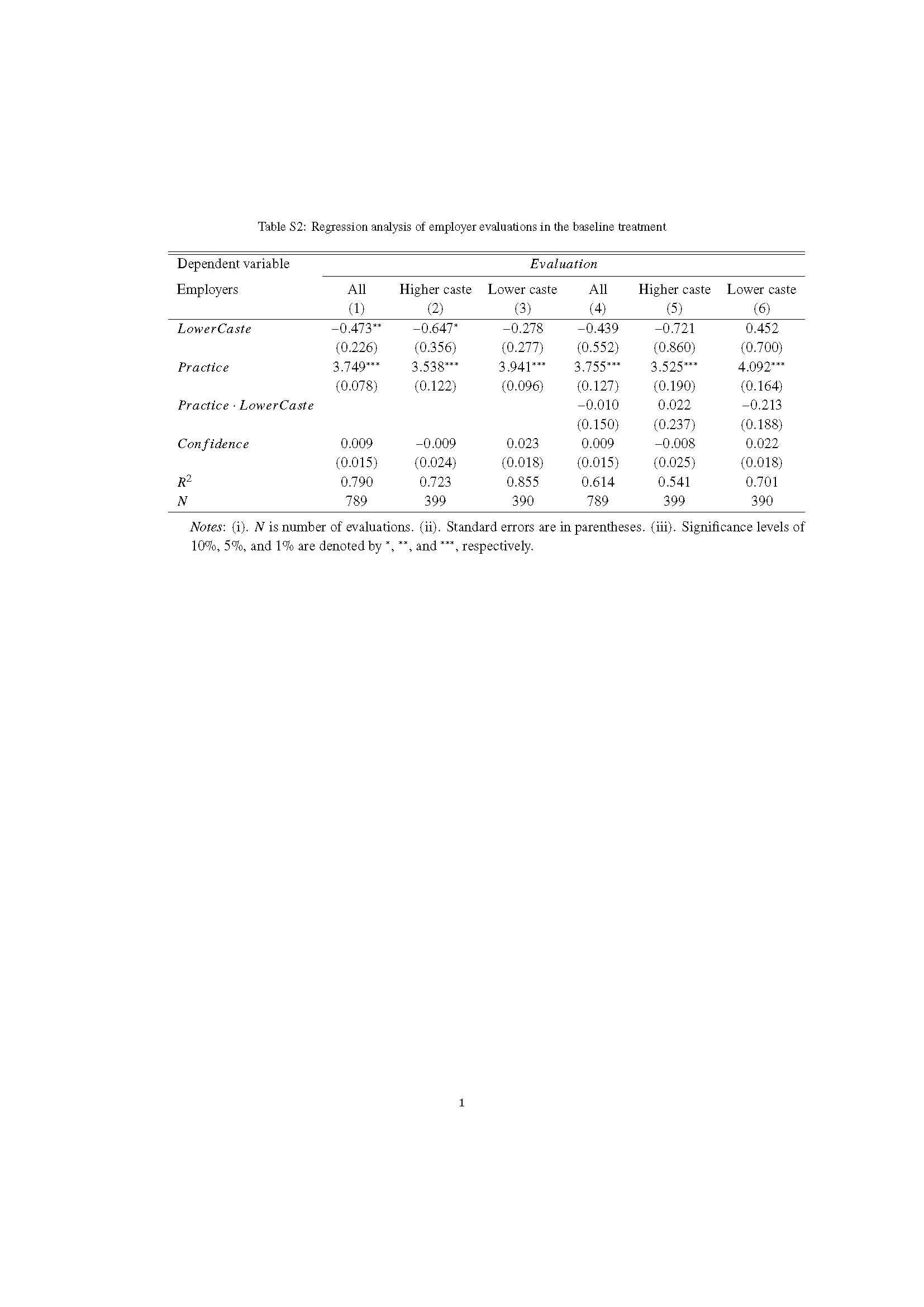

Supplement: S2 Table — (PNG) [file pone.0327299.s002.png]

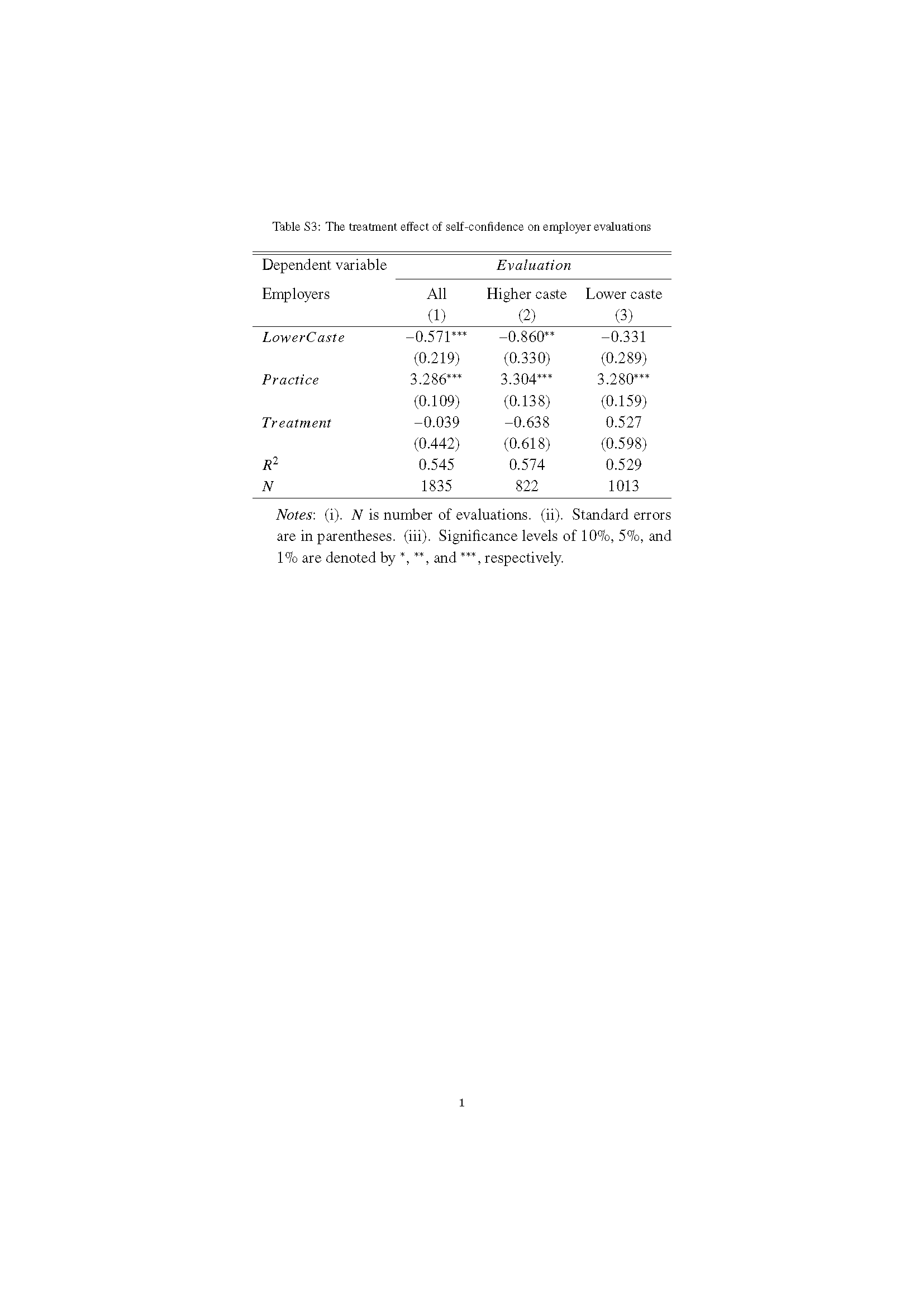

Supplement: S3 Table — (PNG) [file pone.0327299.s003.png]

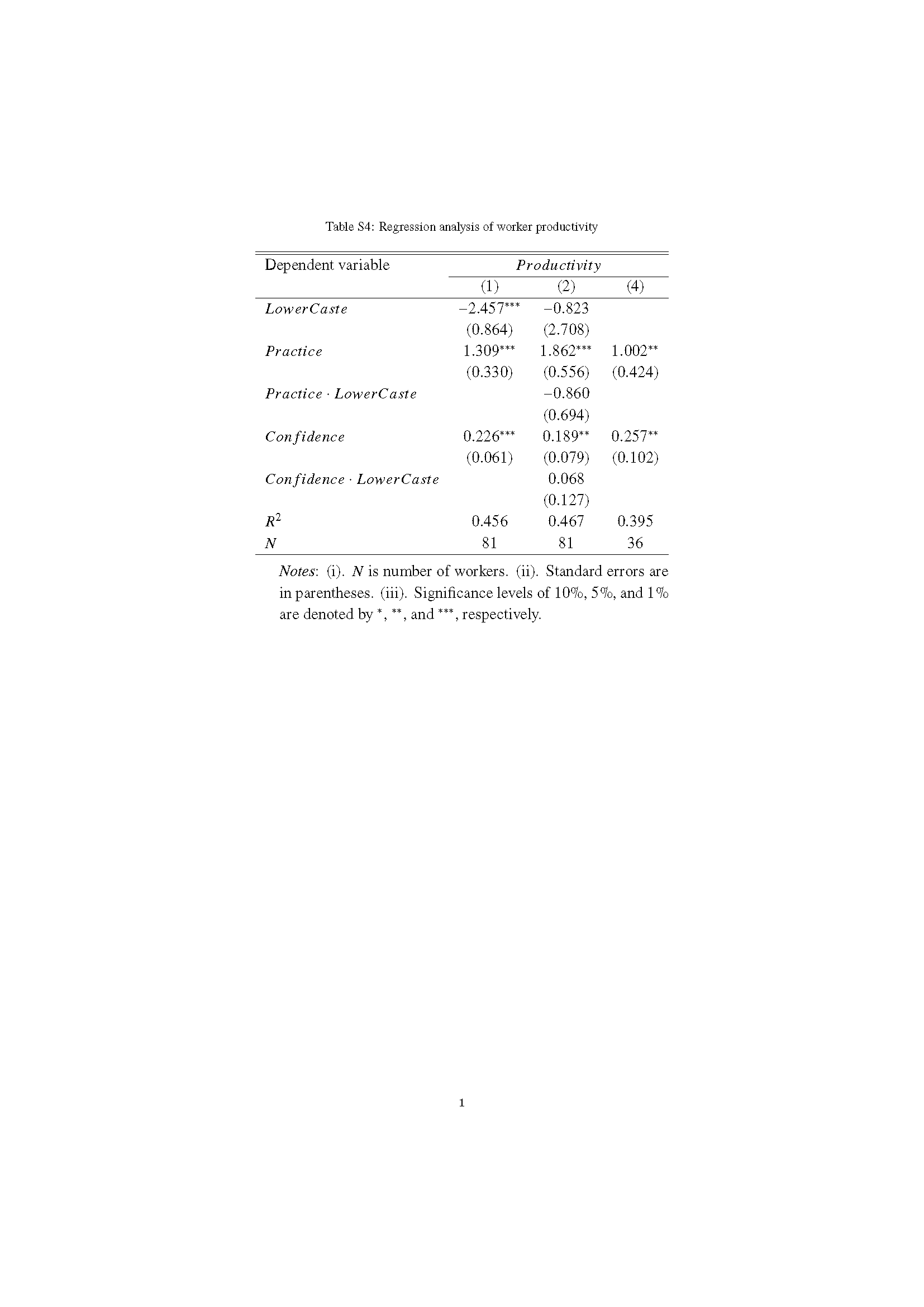

Supplement: S4 Table — (PNG) [file pone.0327299.s004.png]
