# Supplementary material for: Promoting caste equality in the labor market: The role of self-confidence
Source: PLoS One. 2025 Jul 31;20(7):e0327299. doi: 10.1371/journal.pone.0327299 (PMC12312929; doi:10.1371/journal.pone.0327299)
Supplement: S1 File — (PDF) [file pone.0327299.s005.pdf]

Instructions and Questionnaire

Experimental instructions for workers

Page 1

You are taking part in an economic decision making experiment. You have received Rs 100 for participating in this experiment. During this experiment you can earn more money by receiving tokens. The number of tokens that you will receive depends on your decisions. The tokens will be exchanged into Rupees at the end of this experiment. The exchange rate is: 50 tokens = 10 Rupees

In this experiment, there are two roles: The worker and the employer. Today you will play the role of the worker. We will invite a different pool of subjects to play the role of the employer. Your task as the worker is to solve as many character puzzles as possible in a five-minute employment period. For every puzzle that you solve in the five-minute employment period, you will receive 50 tokens. For example, if you solve 10 puzzles you will receive 500 tokens which represent 100 Rupees.

As the worker, you will be evaluated by several employers who set your wages. Each employer will see your performance in a one-minute practice period. The employer's task is to estimate as precisely as possible how many puzzles you are able to perform during the five-minute employment period. The employer's earnings will be higher the better he/she predicts your performance.

The employer's evaluation of your puzzle-solving skills can increase your earnings as the worker. Your earnings will increase by the employer's average evaluation of your puzzle-solving skills times 50 tokens. For example, if employers estimate on average that you can solve 5 puzzles, then you would receive 5 times 50 = 250 tokens additionally.

Only go to the next page when you are informed to do so.

Page 2

On this page, you have the opportunity to solve two example puzzles to familiarize yourself with your task. The square with characters on the right differs from the square of characters on the left in two letters. To solve the puzzle, you have to find and circle those two letters.

CHARACTER PUZZLE

Page 3

On the next page, you have a one-minute practice period to solve puzzles. The number of puzzles you solve in the one-minute practice period will be visible to employers who later evaluate you puzzle-solving ability.

Remember, that the higher the employer's average evaluation of your puzzle-solving ability, the higher are your earnings, as the employer's average evaluation will be multiplied by 50 tokens and added to your earnings.

Only go to the next page when you are informed to do so.

Page 4
Please solve the practice puzzles in a one-minute practice period.

CHARACTER PUZZLE

Page 5
Given your performance in the practice period, please answer the following question: *How many puzzles do you think you can solve in the five-minute employment period?*

If your answer matches with the number that you solve in the actual five-minute employment period, you will receive additional 40 tokens.

Your answer:

Page 6
On the next page, you are asked to solve as many puzzles as possible in the following five-minute employment period. You will receive 50 tokens for each solved puzzle. When time is up we will ring a bell and collect the puzzles that you solve.

CHARACTER PUZZLE

Page 7

Please fill up the following survey:

1. What is your Gender ? Male Female

2. What is your Age?

3. What is your area of study?

4. Are you an undergraduate, Masters, M Phil or PhD student?

5. What is your home state?

6. What category do you belong to: General/Scheduled caste/ Scheduled tribe/creamy OBC category/ non-creamy OBC category?

7. What is the approximate monthly income of your family?

8. What is the percentage of marks you received in class 10?

9. On a scale of 1 to 5, rate how beautiful you consider yourself? 1- not at all and 5- very beautiful

1 2 3 4 5

10. On a scale of 1 to 5, rate how you consider your skin complexion? 1- dark and 5- very fair

1 2 3 4 5

11. On a scale of 1 to 5, rate your confidence level in general in life? 1- not and 5- very confident

1 2 3 4 5

12. What is the occupation of your father?

13. What is the occupation of your mother?

14. Is your father in government sector?

15. What is your future goal?

16. What is the highest education level attained by your father?

17. What is the highest education level attained by your mother?

Experimental instructions for employers

Page 1

You are taking part in an economic decision making experiment. You have received Rs 100 for participating in this experiment. During this experiment you can earn more money by receiving tokens. The number of tokens that you will receive depends on your decisions. The tokens will be exchanged into Rupees at the end of this experiment. The exchange rate is: 50 tokens = 10 Rupees

In this experiment, there are two roles: The worker and the employer. Today you will play the role of the employer. We have invited a different pool of subjects to play the role of the worker. The workers participated in a five-minute employment period. Their task was to solve character puzzles. Below is an example of the puzzles they solved. The square with characters on the right differs from the square of characters on the left in two letters. To solve the puzzle, the worker had to find and circle those two letters.

CHARACTER PUZZLE

Page 2

Before the workers participated in the five-minute employment period, they performed in a one-minute practice period.

Baseline: As the employer, you have to evaluate the performance of 10 workers in the five-minute task period. We will provide you with each worker's resume displaying the worker's performance in the one-minute practice period and caste.

Confidence: As the employer, you have to evaluate the performance of 10 workers in the five-minute task period. We will provide you with each worker's resume displaying the worker's performance in the one-minute practice period, caste, and self-evaluation that the worker predicts he/she could solve in the five-minute task period.

For each worker, you will receive 300 tokens if you predict the worker's performance in the five-minute task precisely. If your estimate is off by X puzzles for this worker, you will receive 300 tokens minus X times 10 tokens. For example: If you predict that the worker solved 5 puzzles and he or she solved actually 3, your earnings are 280 tokens (300 tokens minus 2 times 10 tokens). Similarly, if you predict that the worker solved 5 puzzles and he or she

solved actually 8, you earn earns 270 tokens (300 tokens minutes 3 times 10 tokens).

Your evaluation of the worker's puzzle-solving skills can increase that worker's earnings. Each worker will be evaluated by several employers, and the worker's earnings will increase by the average evaluation of all employers' times 50 tokens.

Page 3
Please evaluate the performance of the following 10 workers in the five-minute task period.

Page 4

Please fill up the following survey:

1. What is your Gender ? Male Female

2. What is your Age?

3. What is your area of study?

4. Are you an undergraduate, Masters, M Phil or PhD student?

5. What is your home state?

6. What category do you belong to: General/Scheduled caste/ Scheduled tribe/creamy OBC category/ non-creamy OBC category?

7. What is the approximate monthly income of your family?

8. What is the percentage of marks you received in class 10?

9. On a scale of 1 to 5, rate how beautiful you consider yourself? 1- not at all and 5- very beautiful

1 2 3 4 5

10. On a scale of 1 to 5, rate how you consider your skin complexion? 1- dark and 5- very fair

1 2 3 4 5

11. On a scale of 1 to 5, rate your confidence level in general in life? 1- not and 5- very confident

1 2 3 4 5

12. What is the occupation of your father?

13. What is the occupation of your mother?

14. Is your father in government sector?

15. What is your future goal?

16. What is the highest education level attained by your father?

17. What is the highest education level attained by your mother?
